# Inhibition of NKCC1 Ameliorates Anxiety and Autistic Behaviors Induced by Maternal Immune Activation in Mice

**DOI:** 10.3390/cimb46030121

**Published:** 2024-02-28

**Authors:** Hai-Long Zhang, Shufen Hu, Shu-Ting Qu, Meng-Dan Lv, Jun-Jun Wang, Xin-Ting Liu, Jia-He Yao, Yi-Yan Ding, Guang-Yin Xu

**Affiliations:** Jiangsu Key Laboratory of Neuropsychiatric Diseases, Institute of Neuroscience, Suzhou Medical College of Soochow University, Medical Center of Soochow University, Suzhou 215123, China; sfhu@suda.edu.cn (S.H.); 20215232078@stu.suda.edu.cn (S.-T.Q.); 20214254011@stu.suda.edu.cn (M.-D.L.); wjxt@gdmu.edu.cn (J.-J.W.); xtliu_egret@163.com (X.-T.L.); 2030504097@stu.suda.edu.cn (J.-H.Y.); duanzi2003@163.com (Y.-Y.D.)

**Keywords:** autism, social behavior, maternal immune activation, poly(I:C), NKCC1

## Abstract

Autism spectrum disorder (ASD) is thought to result from susceptibility genotypes and environmental risk factors. The offspring of women who experience pregnancy infection have an increased risk for autism. Maternal immune activation (MIA) in pregnant animals produces offspring with autistic behaviors, making MIA a useful model for autism. However, how MIA causes autistic behaviors in offspring is not fully understood. Here, we show that NKCC1 is critical for mediating autistic behaviors in MIA offspring. We confirmed that MIA induced by poly(I:C) infection during pregnancy leads to autistic behaviors in offspring. We further demonstrated that MIA offspring showed significant microglia activation, excessive dendritic spines, and narrow postsynaptic density (PSD) in their prefrontal cortex (PFC). Then, we discovered that these abnormalities may be caused by overexpression of NKCC1 in MIA offspring’s PFCs. Finally, we ameliorated the autistic behaviors using PFC microinjection of NKCC1 inhibitor bumetanide (BTN) in MIA offspring. Our findings may shed new light on the pathological mechanisms for autism caused by pregnancy infection.

## 1. Introduction

Autism Spectrum Disorder (ASD) is a neurodevelopmental disorder that develops in infancy and early childhood, clinical symptoms include language delay, social impairment, and stereotyped behavior [1,2,3,4,5,6]. The increasing clinical prevalence of ASD in recent years has attracted widespread attention worldwide [3]. The current international prevalence is 1 in 44, and there are no effective therapeutic drugs available [7]. The main causes of ASD include genetic mutations and pregnancy infections [8,9,10], and maternal immune activation (MIA) caused by pregnancy infection with the RNA virus analogue poly(I:C) is a common model of ASD in animals [11,12,13]; however, the pathogenesis of ASD caused by pregnancy infection is not fully understood.

The recent outbreak of COVID-19, an RNA virus that has infected millions of pregnant women worldwide, may further increase the incidence of ASD [14,15,16]. Since ASD is the leading psychiatric disabling disorder in children, it imposes a huge economic burden on families and society [17]. Therefore, the study of the pathogenesis of ASD caused by pregnancy infection has significant clinical translational implications. ASD is influenced by susceptibility genes and environmental exposures, and pregnancy infection plays an important role in the pathogenesis of ASD, with genetically susceptible individuals exposed to pregnancy infection being more likely to develop ASD [13]. Studies have shown that maternal immune inflammation, rather than direct fetal infection, is responsible for the increased incidence of autism in offspring after pregnancy infection [18,19]. Influenza infection during pregnancy leads to autistic disorders in offspring, but the virus has not been detected in fetuses [20,21]. Importantly, injection of the RNA virus analogue poly(I:C) into pregnant mice results in behavioral and histological abnormalities in MIA offspring [22,23,24]. Previous studies have shown that prenatal poly(I:C) exposure leads to microglia activation in offspring [25,26,27]. However, it remains unknown as to how maternal immune activation by pregnancy infection disrupts offspring brain development and function.

The pathogenesis of ASD involves an imbalance between excitation and inhibition. In neurons, the Cl^−^ importer NKCC1 (Na-K-Cl cotransporter 1) is counterbalanced by the Cl^−^ extruder KCC2 (K-Cl cotransporter 2) [28,29,30]. NKCC1 and KCC2 serve as the primary regulators of the transmembrane Cl^−^ gradient in neurons [31]. These cotransporters play a crucial role in the GABA switch during early development and contribute to the inhibitory actions of the GABA_A_ receptor (GABA_A_R) [32,33,34]. The shift from GABA excitatory to inhibitory effects is attributed to the developmental switch in the expression of NKCC1 and KCC2 proteins [35]. Despite the notable positive effects of the NKCC1 inhibitor BTN on the pathophysiology of ASD [36,37,38,39,40,41], the specific mechanism by which it operates remains unclear. BTN functions as an antagonist to neuronal NKCC1, aiming to reduce intracellular Cl^−^ levels and facilitate GABAergic-mediated hyperpolarization. The administration of BTN as an anti-ASD agent is based on the rationale that increased NKCC1 expression, unbalanced by the efflux activity of KCC2, contributes to the development of ASD. However, it is unclear whether NKCC1 expression is increased in ASD caused by pregnancy infection, and whether bumetanide could alleviate MIA-induced autistic behaviors.

Therefore, we propose the hypothesis that maternal immune activation caused by pregnancy infection with the RNA virus analogue poly(I:C) leads to microglia activation and NKCC1 overexpression in the offspring brain, resulting in abnormal brain development and autistic behaviors.

## 2. Materials and Methods

### 2.1. Experimental Animals

C57BL/6J mice at 8 weeks old or offspring mice at 4 weeks old were used in experiments unless otherwise described. Mice were mated overnight, and the presence of a vaginal plug marked that day as embryonic day 0 (E0). Pregnant females were not disturbed, until E12.5 when they were given intraperitoneal injections of 20 mg/kg poly(I:C) (potassium salt; Sigma, St. Louis, MO, USA) freshly dissolved in saline. Animals were housed in rooms at 22 °C and 50% humidity in a 12/12 h light/dark cycle and with food and water available ad libitum. All experimental procedures were approved by the Institutional Animal Care and Use Committee of Soochow University (SYXK 2022-0043). All animal experiments followed the ARRIVE guidelines.

### 2.2. Western Blotting

Previous studies [42] have detailed the procedure for performing Western blotting. The quantification of NKCC1/KCC2 expression in the PFCs of 4-week-old mice was accomplished through Western blotting analysis. To extract the PFC tissues, a RIPA lysis buffer (Shanghai Macklin Biochemical Co., Ltd., Shanghai, China) was employed. Separation of 30 μg of total protein was achieved using 10% SDS-PAGE, followed by transfer to PVDF membranes. Subsequently, these membranes were incubated in a TBS buffer consisting of 0.1% Tween-20 and 3% BSA at room temperature for 1 h prior to overnight incubation with a primary antibody at 4 °C. After washing, the membranes were incubated at room temperature for 1 h with an HRP-conjugated secondary antibody in the same TBS buffer. Visualization of immunoreactive bands was accomplished through ChemiDoc^TM^ XRS + Imaging System (BIO-RAD, Hercules, CA, USA) utilizing enhanced chemiluminescence (NCM Biotech, Suzhou, China), and the analysis was performed with Image J 1.8.0 (NIH, Bethesda, MD, USA). The primary antibodies employed were NKCC1 (1:1000, Thermo Fisher, Waltham, MA, USA; PA5-98154), KCC2 (1:1000, Cell Signaling, Danvers, MA, USA; 94725), and anti-α-tubulin (1:10,000, Cell Signaling, 3873). There were six offspring in each of the saline and poly(I:C) groups.

### 2.3. Immunofluorescence Staining

For fixation of PFC slices obtained from 4-week-old mice, a solution containing 4% PFA was utilized. Subsequently, permeabilization was carried out using 0.3% Triton-X 100 and 5% BSA in PBS, followed by overnight incubation at 4 °C with the primary antibody. Washing steps were performed thrice with PBS before incubation with Goat anti-Mouse Alexa Fluor 488-conjugated secondary antibody (1:500, Thermo Fisher, Waltham, MA, USA; A-11029) for 1 h at room temperature. To mount the samples, Vectashield mounting medium (Vector, Newark, CA, USA) was employed, and images were captured using a Leica TCS SP8 confocal microscope (Leica, Wetzlar, Germany). The primary antibody employed was mouse anti-Iba1 (1:500, Wako, Richmond, VA, USA; NCNP24). There were six offspring in each of the saline and poly(I:C) groups.

### 2.4. Golgi Staining

To prepare the brains of 4-week-old mice, the standard Golgi-Cox impregnation technique in combination with the FD Rapid GolgiStain Kit (NeuroTechnologies, Vilnius, Lithuania) was employed. Coronal sections of 100 µm were collected serially. From the PFC, a representative sampling approach was adopted to trace a total of 10 cells per mouse, resulting in a final count of 30 cells per group. There were six offspring in each of the saline and poly(I:C) groups.

### 2.5. Electron Microscopy

Electron microscopy was performed as described in previous studies [43]. The mice at 4 weeks old were deeply anesthetized with an overdose of isofluorane and transcardially perfused with PBS (pH 7.4) followed by ice-cold 4% paraformaldehyde (PFA) in phosphate buffer (pH 7.4). The brains were removed, dissected PFC slices, and kept overnight in PFA 4% for post-fixation. Then, they were transferred to a 4% glutaraldehyde solution and stored at 4 °C for 3 days. After that, the samples underwent two 20 min washes in 7.5% sucrose and 0.1 M sodium cacodylate buffer, followed by post-fixation in 1% osmium tetroxide with an initial microwave treatment of 6 min. The next step involved two 20 min washes in 0.11 M veronal acetate buffer. Subsequently, the samples were enblock stained with 1% uranyl acetate in distilled water for 1 h and underwent two more 20 min washes in 0.11 M veronal acetate buffer. The dehydration process included 20 min treatments in ethanol dilutions (70%, 95%, 100%), with an initial microwave treatment of 2 min. The samples were then treated with propaline oxide twice for 20 min and impregnated with a 50:50 mixture of propaline oxide and Epon resin overnight at 4 °C, with an initial microwave treatment for 3 min. Next, the samples were impregnated with 100% Epon resin, with three changes that lasted 2 h each, and an initial microwave treatment of 3 min for each change. The tissue samples were embedded in molds and incubated at 60 °C for 48 h. Following that, semi-thin sections (1 μm) were cut using a Leica UltraCut ultramicrotome and stained with Toluidine stain (0.8%). Thin PFC sections (100 nm) were obtained from these, and they were cut on an UltraCut, placed on 200 mesh Metaxaform Copper Rhodium grids, and post-stained with 2% uranyl acetate in distilled water for 15 min and Sato’s Lead citrate stain for 7 min. Finally, the grids were examined using a Philips (FEI) CM 12 transmission electron microscope at 40,000× magnification. The images were captured using an AMT Vue system with an ORCA HR high-resolution digital camera (7 megapixels) and a Hamamatsu DCAM board for acquisition, using AMT Image Capture Engine software version 600f. The saved images were in an 8-bit TIFF format with a resolution of 7.5 megapixels. PSD measurements were conducted using Image J software (1.8.0) from NIH, USA. There were six offspring in each of the saline and poly(I:C) groups.

### 2.6. Microinjection of Bumetanide into PFC

The 4-week-old mice were anesthetized using isoflurane and head-fixed in a stereotaxic device (RWD life science, San Diego, CA, USA). The mice were implanted bilaterally with stainless steel guide cannulae (RWD life science) in their PFCs, using the following coordinates relative to bregma: anteroposterior (AP) + 1.9 mm, mediolateral (ML) ± 0.4 mm, dorsoventral (DV) − 1.5 mm. In PFC infusion experiments, we tilted 30° in canula surgery to avoid collision. To administer the drug, we connected the infusion cannulae to a microsyringe, which was driven by a microinfusion pump (KDS 310, KD Scientific, Holliston, MA, USA) through PE20 tube. An internal cannula extending 1 mm beyond the guides were used for drug infusions. We used an infusion pump to perform a daily microinjection of bumetanide into their PFCs at a rate of 1 μL/min. After injection, the needle was left in place for an additional 10 min to allow the drug to diffuse adequately. The bumetanide (TOCRIS) was dissolved in saline (0.1 M), and we injected 5 μL of the stock solution into the two-side PFCs using the infusion cannulae.

### 2.7. Open Field Test

At the age of 4 weeks old, the mice were placed in a chamber measuring 40 cm × 40 cm × 35 cm and observed for a duration of 10 min to monitor their movement. The any-maze video tracking system (manufactured by Stoelting, Wood Dale, IL, USA) was used to measure the overall distance covered by the mice in the open field. Following each test session, the chamber was meticulously cleaned using 75% ethanol and thoroughly dried. There were 12 offspring in each of the saline, poly(I:C), poly(I:C) + saline, and poly(I:C) + BTN groups.

### 2.8. Elevated Plus Maze

The protocol was followed as described [44]. A white plastic material was utilized to construct the elevated plus maze, which comprised of four arms (two arms devoid of walls and two arms enclosed by 15 cm high walls), measuring 30 cm in length and 5 cm in width. The maze was elevated 40 cm above the floor, as it was firmly attached to robust metal legs. To monitor activity, a digital camera was suspended from the ceiling, and recordings were made. After each test, the maze was thoroughly cleaned using 75% ethanol. Individual mice at the age of 4 weeks old were placed at the center of the maze, facing the enclosed arms, and their behavior was recorded for a duration of 5 min. The time spent on both the open and closed arms was documented and subsequently analyzed through the implementation of the any-maze video tracking system (Stoelting, Wood Dale, IL, USA). There were 12 offspring in each of the saline, poly(I:C), poly(I:C) + saline, and poly(I:C) + BTN groups.

### 2.9. Grooming Behavior

In this investigation, we examined the grooming actions exhibited by mice at the age of 4 weeks. Each mouse was housed alone and monitored for a continuous duration of 24 h. The monitoring period consisted of 12 h of daylight (700 lx) followed by 12 h of low-intensity red light at night (2 lx). The observational data regarding grooming behaviors were meticulously collected from 19:00 to 20:00, precisely one hour subsequent to the initiation of the dark phase. To analyze this specific timeframe, we employed HomeCageScan software 2.0 (Clever Sys, Reston, VA, USA) and measured the total duration mice spent engaging in grooming behaviors. Grooming actions encompassed face-wiping, scratching or rubbing of the head and ears, and comprehensive body grooming. There were 12 offspring in each of the saline, poly(I:C), poly(I:C) + saline, and poly(I:C) + BTN groups.

### 2.10. Social Ability and Novelty

The three-chamber box, which measured 60 cm × 40 cm × 25 cm, was utilized to assess the social behavior of mice at age 4 weeks old. In each end chamber, a transparent Plexiglas cylinder was present. One of the cylinders accommodated a social mouse that matched the age and sex of the mice being tested, while the other cylinder remained unoccupied. Initially, the test mice were positioned in the central chamber and allowed to explore the chambers freely for a duration of 10 min. It is important to note that the chambers consisted of two empty cylinders. To appraise social ability, an extra 5 min were allotted for the mice to investigate the chambers containing a social cylinder (designated as Mouse, M) and a non-social cylinder (classified as Empty, E). Following a time interval of 30 min, the mice proceeded to undergo a social novelty test. This particular examination granted the mice 5 min to explore the chambers that housed cylinders containing a familiar mouse (identified as F) and a novel mouse (referred to as N). The any-maze video tracking system (manufactured by Stoelting, Wood Dale, IL, USA) was utilized to record video sessions, and subsequent analysis of the recordings enabled examination of the time spent in proximity to the social cylinder and non-social cylinder, as well as the familiar cylinder and novel cylinder. To ensure hygiene, both the box and cylinders were meticulously cleaned with 75% ethanol and thoroughly dried following the completion of each testing session. There were 12 offspring in each of the saline, poly(I:C), poly(I:C) + saline, and poly(I:C) + BTN groups.

### 2.11. Statistical Analysis

All the data were shown as mean value ± SEM. Comparisons between two groups were made using a two-sided *t* test. Comparisons between three or more groups were made using one-way ANOVA followed by Tukey’s multiple comparisons test. Data on social ability and novelty were analyzed using two-way ANOVA followed by Tukey’s multiple comparisons test. Statistically significant differences were indicated as follows: *** *p* < 0.001, ** *p* < 0.01 and * *p* < 0.05. The statistical analysis was performed with GraphPad Prism 9.

## 3. Results

### 3.1. MIA Offspring Exhibit Anxiety and Autistic Behaviors

**Figure 1:**
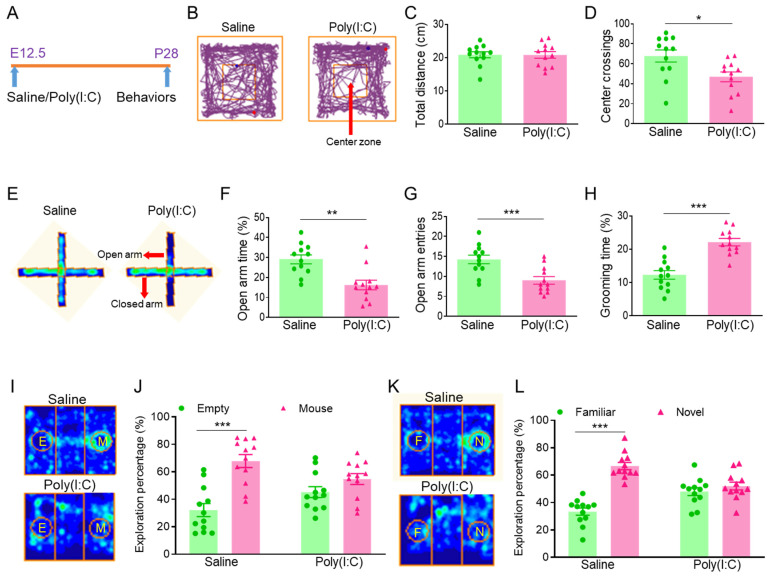
Poly(I:C) injection during pregnancy led to anxiety and autistic behaviors in MIA offspring. (**A**) At embryonic day 12.5 (E12.5), poly(I:C) was injected intraperitoneally to construct a mouse autism model caused by maternal immune activation (MIA), and MIA offspring were tested behaviorally at postnatal day 28 (P28). (**B**) Trajectory map of open field. (**C**) Total distance of open field. (**D**) Number of center crossings of open field, * *p* < 0.05, n = 12. (**E**) Heat map of elevated plus maze. (**F**) Stay time in the open arm, ** *p* < 0.01, n = 12. (**G**) Number of entries in the open arm, *** *p* < 0.001, n = 12. (**H**) Grooming percentage during 1 h beginning at the initiation of the dark cycle, *** *p* < 0.001, n = 12. (**I**) Heat map of three-chamber social ability. (**J**) Preference for the mouse (M) cylinder in the saline group, Column Factor, F (1, 44) = 26.68, *** *p* < 0.001, n = 12. No preference for empty (E) and mouse (M) cylinders in the poly(I:C) group. (**K**) Heat map of three-chamber social novelty. (**L**) Preference for the novel (N) mouse in the saline group, Column Factor, F (1, 44) = 46.25, *** *p* < 0.001, n = 12. No preference for familiar (F) and novel (N) mice in the poly(I:C) group.

To investigate pathogenesis of ASD caused by pregnancy infection, we constructed a mouse autism model for MIA caused by pregnancy infection with the RNA virus analogue poly(I:C) [11,12,13]. Pregnant females were given intraperitoneal injections of 20 mg/kg poly(I:C) or saline at embryonic day 12.5 (E12.5), their offspring at age 4 weeks were tested for their emotional state and social behavior (Figure 1A). To detect locomotor activity and anxiety behavior in the offspring, we tested the offspring in an open field (Figure 1B). The results showed that the total distances are similar (Figure 1C), but the number of center crossings was decreased in the poly(I:C) group (Figure 1D), compared to the saline group. We further performed an elevated plus maze test to examine the anxiety behavior of the offspring (Figure 1E). The results displayed that the stay time and number of entries in the open arm was reduced in the poly(I:C) group (Figure 1F,G), compared to the saline group. Next, we examined the self-grooming behavior of the offspring. The grooming behavior significantly increased in the poly(I:C) group (Figure 1H), compared to the saline group. In addition, we explored the offspring social behavior using a three-chamber social test. In a three-chamber social ability test (Figure 1I), the saline offspring had a preference for the mouse (M) cylinder, but the poly(I:C) offspring had no preference for empty (E) and mouse (M) cylinders (Figure 1J). The result suggested that the social ability of the poly(I:C) offspring was impaired. In the three-chamber social novelty test (Figure 1K), the saline offspring had a preference for the novel (N) mouse, but the poly(I:C) offspring had no preference for familiar (F) and novel (N) mice (Figure 1L). The result suggested that the social novelty of the poly(I:C) offspring was declined. Overall, the poly(I:C) offspring exhibited anxiety and autistic behaviors.

### 3.2. MIA Offspring Display Microglia Activation in PFC

**Figure 2:**
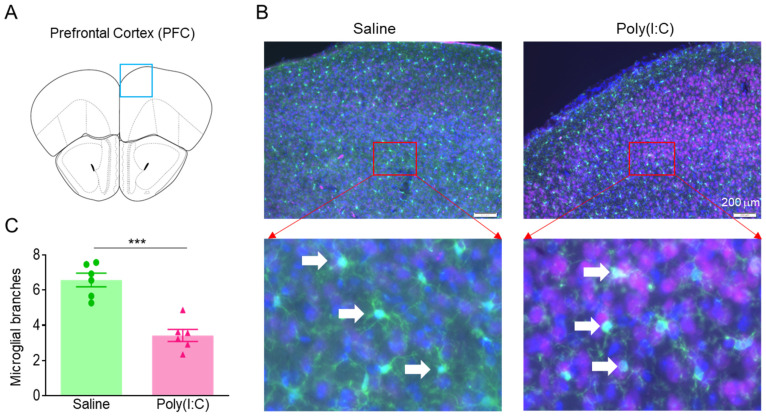
Poly(I:C) injection during pregnancy caused microglia activation in PFC of MIA offspring. (**A**) Diagram to show the mouse PFC coronal section. The blue square represents the staining brain region (**B**) Representative image of microglia staining in PFC of offspring. Images were taken at 10× magnification (upper panel) and 40× magnification (lower panel). The white arrows represent microglia. (**C**) Number of microglial branches in PFC of offspring, *** *p* < 0.001, n = 6.

To investigate whether pregnancy infection causes microglia activation in the PFCs of MIA offspring. The PFC slices from 4-week-old offspring were stained with microglia marker Iba1 (Figure 2A,B). The number of microglial branches in the PFCs of MIA offspring was reduced, indicating microglia activation in the PFCs of MIA offspring (Figure 2B,C).

### 3.3. MIA Offspring Show Excessive Dendritic Spines and Narrow PSD Zone in PFC

**Figure 3:**
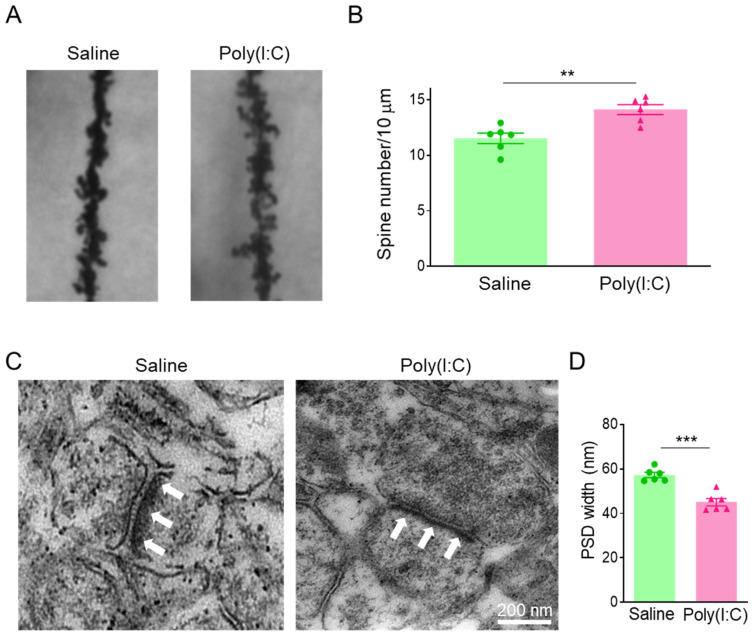
Poly(I:C) injection during pregnancy induced excessive dendritic spines and narrow PSD zone in PFC of MIA offspring. (**A**) Representative Golgi staining in PFC of offspring. (**B**) Excessive dendritic spines in PFC of MIA offspring, ** *p* < 0.01, n = 6. (**C**) Representative electron microscopy in PFC of offspring. (**D**) Narrow PSD zone in PFC of MIA offspring, *** *p* < 0.001, n = 6.

To investigate whether pregnancy infection causes abnormal dendritic spines and synaptic development in PFCs of MIA offspring, the PFC slices from 4-week-old offspring had Golgi staining performed on them. We found an excessive increase in PFC dendritic spines of MIA offspring (Figure 3A,B), compared to the saline group. We further examined the development of the postsynaptic dense (PSD) zone in the PFCs of MIA offspring. The results showed that the PSD zone became thinner in the PFCs of MIA offspring (Figure 3C,D), compared to the saline group.

### 3.4. MIA Offspring Demonstrate Overexpression of NKCC1 in PFC

**Figure 4:**
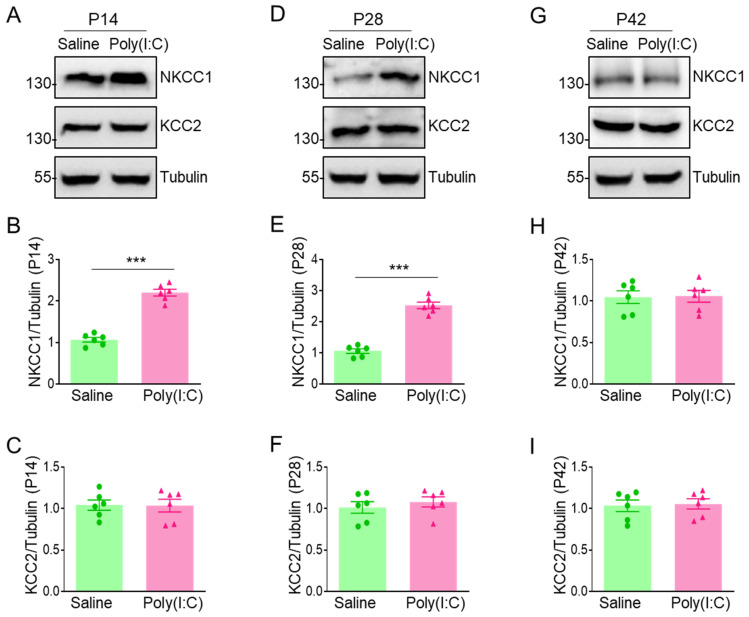
Poly(I:C) injection during pregnancy resulted in overexpression of NKCC1 in PFC of MIA offspring. (**A**) Representative image of NKCC1/KCC2 expression in PFC of offspring at P14. (**B**) Quantification of NKCC1/Tubulin in panel (**A**), *** *p* < 0.001, n = 6. (**C**) Quantification of KCC2/Tubulin in panel (**A**), n = 6. (**D**) Representative image of NKCC1/KCC2 expression in PFC of offspring at P28. (**E**) Quantification of NKCC1/Tubulin in panel (**D**), *** *p* < 0.001, n = 6. (**F**) Quantification of KCC2/Tubulin in panel (**D**), n = 6. (**G**) Representative image of NKCC1/KCC2 expression in PFC of offspring at P42. (**H**) Quantification of NKCC1/Tubulin in panel (**G**), n = 6. (**I**) Quantification of KCC2/Tubulin in panel (**G**), n = 6.

Excitation/inhibition imbalance contributes to the pathogenesis of ASD, which is involved in the developmental shift of NKCC1 and KCC2 expression [35]. To investigate whether NKCC1 expression is changed in ASD caused by pregnancy infection. The expression of NKCC1/KCC2 in the PFCs from offspring were quantified using Western blotting. These results showed that NKCC1 was elevated at P14 and P28 but not P42 in the PFCs of MIA offspring, but KCC2 was not significantly different at P14, P28, and P42 in the PFCs of MIA offspring (Figure 4), compared to the saline group.

### 3.5. Inhibition of NKCC1 Ameliorates Anxiety and Autistic Behaviors in MIA Offspring

**Figure 5:**
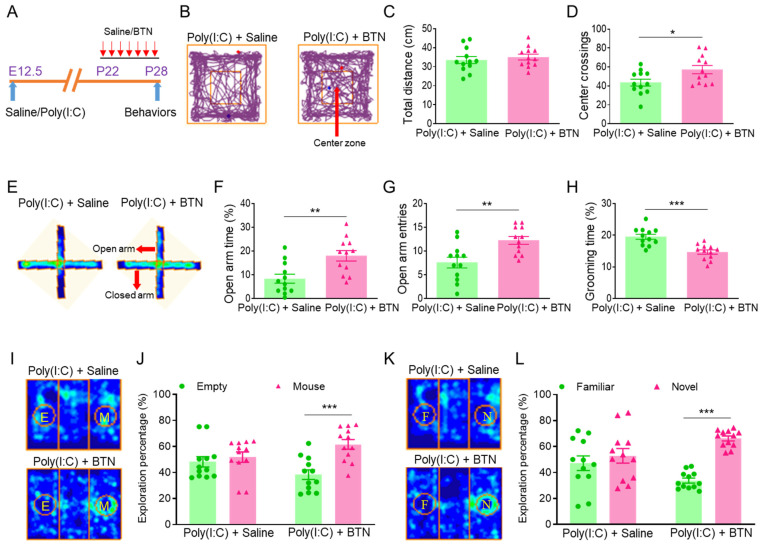
NKCC1 inhibitor bumetanide ameliorated anxiety and autistic behaviors in MIA offspring. (**A**) The MIA offspring were injected with NKCC1 inhibitor bumetanide (BTN) in the PFC at P22-28, and behavioral tests were performed after the last PFC microinjection of BTN at P28. (**B**) Trajectory map of open field. (**C**) Total distance of open field. (**D**) Number of center crossings of open field, * *p* < 0.05, n = 12. (**E**) Heat map of elevated plus maze. (**F**) Stay time in the open arm, ** *p* < 0.01, n = 12. (**G**) Number of entries in the open arm, ** *p* < 0.01, n = 12. (**H**) Grooming percentage during 1 h beginning at the initiation of the dark cycle, *** *p* < 0.001, n = 12. (**I**) Heat map of three-chamber social ability. (**J**) No preference for empty (E) and mouse (M) cylinders in the poly(I:C) + saline group. Preference for the mouse (M) cylinder in the poly(I:C) + BTN group, Column Factor, F (1, 44) = 12.13, *** *p* < 0.001, n = 12. (**K**) Heat map of three-chamber social novelty. (**L**) No preference for familiar (F) and novel (N) mice in the poly(I:C) + saline group. Preference for the novel (N) mouse in the poly(I:C) + BTN group, Column Factor, F (1, 44) = 20.73, *** *p* < 0.001, n = 12.

To investigate whether overexpression of NKCC1 contributes to anxiety and autistic behaviors in MIA offspring, we used NKCC1 inhibitor BTN to test anxiety and autistic behaviors in MIA offspring. The MIA offspring were injected with NKCC1 inhibitor BTN or saline in their PFCs at P22-28, and behavioral tests were performed after the last PFC microinjection of BTN at P28 (Figure 5A). To detect locomotor activity and anxiety behavior in the offspring, we tested the offspring in an open field (Figure 5B). The results showed that the total distances were similar (Figure 5C), but the number of center crossings was increased in the poly(I:C) + BTN group (Figure 5D), compared to the poly(I:C) + saline group, and both poly(I:C) + saline and poly(I:C) + BTN were injected in their PFCs. We further performed an elevated plus maze test to examine the anxiety behavior of the offspring (Figure 5E). The results displayed that the stay time and number of entries in the open arm was improved in the poly(I:C) + BTN group (Figure 5F,G), compared to the poly(I:C) + saline group. Next, we examined the self-grooming behavior of the offspring. The grooming behavior significantly decreased in the poly(I:C) + BTN group (Figure 5H), compared to the poly(I:C) + saline group. In addition, we explored the offspring social behavior using a three-chamber social test. In the three-chamber social ability test (Figure 5I), the poly(I:C) + saline offspring had no preference for empty (E) and mouse (M) cylinders, but the poly(I:C) + BTN offspring had a preference for the mouse (M) cylinder (Figure 5J). The result suggested that the social ability of the poly(I:C) + BTN offspring was improved. In three-chamber social novelty test (Figure 5K), the poly(I:C) + saline offspring had no preference for familiar (F) and novel (N) mice, but the poly(I:C) + BTN offspring had a preference for the novel (N) mouse (Figure 5L). The result suggested that the social novelty of the poly(I:C) + BTN offspring was raised. Overall, NKCC1 inhibition ameliorated anxiety and autistic behaviors in MIA offspring.

## 4. Discussion

**Figure 6:**
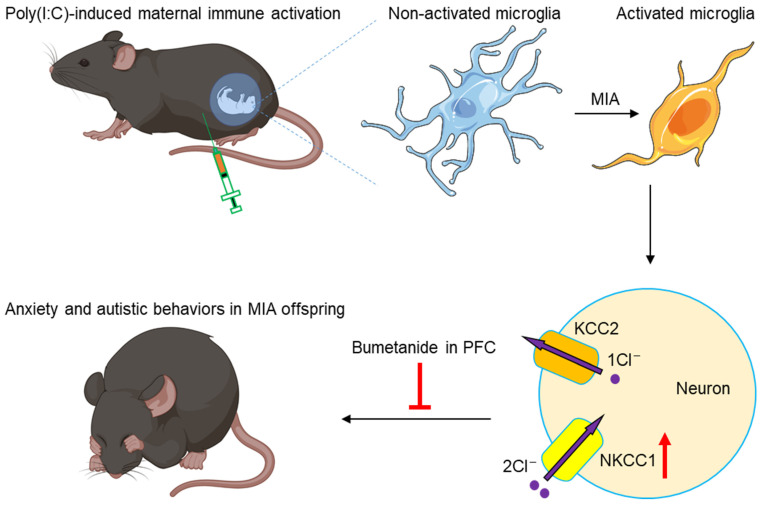
Working model. Maternal immune activation caused by pregnancy infection with the RNA virus analogue poly(I:C) leads to microglia activation and NKCC1 overexpression in the offspring PFC, resulting in excessive dendritic spines and narrow PSD zones and autistic behaviors. Continuous NKCC1 inhibition ameliorated anxiety and autistic behaviors in MIA offspring.

In summary, maternal immune activation caused by pregnancy infection with the RNA virus analogue poly(I:C) leads to microglia activation and NKCC1 overexpression in the offspring’s PFC, resulting in excessive dendritic spines and narrow PSD zones and autistic behaviors. Continuous NKCC1 inhibition ameliorated anxiety and autistic behaviors in MIA offspring (Figure 6).

We chose the MIA model of ASD for several reasons. First, MIA is a classical pregnancy infection model of ASD [11,12,13], which differs from the genetic mutation model of ASD; its pathogenesis is not well understood. Second, with the COVID-19 pandemic, millions of pregnant women were infected, which may further increase the prevalence of ASD [14,15,16]. Coronavirus is an RNA virus, while the MIA model uses RNA virus analogue poly(I:C), and there is a certain similarity between the two pathogens. Pregnant females were given intraperitoneal injections of 20 mg/kg poly(I:C) at embryonic day 12.5 (E12.5), the results suggest that the MIA offspring show anxiety and autistic behaviors, which is consistent with previous studies [11,12,13]. The mode of infection with intraperitoneal injection, the infectious dose of 20 mg/kg poly(I:C), and the duration of infection at E12.5 were the limiting factors leading to the autistic behavior exhibited by the MIA offspring [45]. Because the mode, dose, and week of gestation of coronavirus infections vary among pregnant women, the association between coronavirus infections in pregnant women and the increased incidence of ASD remains to be further investigated. Poly(I:C) is a synthetic double-stranded RNA analog of retroviral genomic dsRNA. When administered to pregnant rodents, it mimics a viral infection during pregnancy and, therefore, serves as a model to investigate the association between MIA and neurodevelopmental disorders such as ASD [11,12,13]. Coronaviruses are a group of related RNA viruses that cause diseases in mammals and birds. They cause respiratory tract infections that can range from mild to lethal. Mild illnesses in humans include some cases of the common cold, while more lethal varieties can cause SARS, MERS and COVID-19. In cows and pigs, they cause diarrhea, while in mice they cause hepatitis and encephalomyelitis [46,47,48]. Poly(I:C) exposure only mimics part of the inflammatory process of coronaviruses invasion of the organism, and poly(I:C) does not have complex functions such as replication and proliferation, nor does it have the special structure of coronaviruses. Thus, pregnancy infection caused by the RNA virus analogue poly(I:C) does not account for the aspects of pregnancy infection attributed to coronaviruses.

Maternal immune activation (MIA) caused by pregnancy infection with the RNA virus analogue poly(I:C) leads to autistic behavior in animals [11,12,13]; however, the pathogenesis of ASD caused by pregnancy infection is elusive. Studies have shown that maternal immune inflammation, rather than direct fetal infection, leads to autistic behavior in offspring [18,19]; the virus has not been detected in fetuses [20,21]. However, it is not clear whether MIA could cause microglia activation, dendritic spine changes, and PSD abnormalities in the PFCs of offspring. Our results have shown that MIA leads to microglia activation, excessive dendritic spines and narrow PSD zones in the PFCs of offspring. However, why maternal immune inflammation causes microglia activation and abnormal synaptic development in the PFCs of offspring needs to be further investigated. We propose that maternal inflammatory factors may enter the fetal brain through the umbilical cord blood circulation, causing microglia activation, neurodevelopmental abnormalities, and autistic behaviors in MIA offspring. We constructed mouse models in which each dam gave birth to roughly 8–12 pups, and we tested only male offspring, providing about 3–6 weaned male offspring per dam. At least 6 dams were constructed per group to meet the number of offspring, including the four groups used in the paper. We randomly selected 12 offspring per group for the experiment (2 offspring were randomly provided from each of the 6 dams), and 2 of each of these offspring were brothers, but all of these offspring were C57BL/6J mice with the same genetic background. It is possible that these two brothers produce the litter effect, which needs to be further investigated with more littermates.

Previous studies have shown that maternal immune activation alters fetal brain development through TLR3 [45], interleukin-6 [11], and Microbiota [12]. Our results have shown that NKCC1 is also important for anxiety and autistic behaviors in the MIA offspring, this is consistent with the findings of the fragile X mice [34]. These results suggest that genetic mutation models and pregnancy infection models may share a common NKCC1 overexpression mechanism for anxiety and autistic behaviors. Notably, NKCC1 overexpression does not start and end at the same time in different autism models, suggesting that the time window for future administration of the NKCC1 inhibitor bumetanide is different and requires precision medicine based on individual specificity. However, further studies are needed to investigate why maternal immune activation causes NKCC1 overexpression in the PFCs of MIA offspring. Poly(I:C) binds to the toll-like receptor 3 (TLR3) in an organism, which results in the release of pro-inflammatory cytokines and initiates the inflammatory cascade in a fashion similar to a viral infection. It is possible that poly(I:C) activates TLR3, which increases IL-6 and mediates NKCC1 overexpression [11,45,49].

In general, as far as we know, this study was the first to show that NKCC1 was elevated in MIA offspring at P14 and P28, and continuous NKCC1 inhibition ameliorated anxiety and autistic behaviors in MIA offspring. Previous studies have shown that MIA in mice only partially mimics symptoms of ASD. The prenatal Poly I:C paradigm appears to be a useful model for studying the effects of maternal immune activation on brain development and behavioral development, but does not necessarily encompass the full range of symptoms of ASD [50]. Our study also had several limitations, including limited samples and focus on only an MIA model. Further studies would use more samples, animal models, advanced methods, and investigate the pathogenesis of ASD caused by pregnancy infection.

## 5. Conclusions

In summary, maternal immune activation leads to microglia activation, excessive dendritic spines, narrow PSD zones, and NKCC1 overexpression in the PFC, and anxiety and autistic behaviors in the offspring. Microinjection of NKCC1 inhibitor bumetanide in PFCs ameliorated anxiety and autistic behaviors in MIA offspring.

## Data Availability

The raw data supporting the conclusions of this article will be made available by the authors, without undue reservation.
